# The use of external event monitoring (web-loop) in the elucidation of symptoms associated with arrhythmias in a general population

**DOI:** 10.1590/S1679-45082014AO2939

**Published:** 2014

**Authors:** Hindalis Ballesteros Epifanio, Marcelo Katz, Melania Aparecida Borges, Alessandra da Graça Corrêa, Fátima Dumas Cintra, Rodrigo Leandro Grinberg, Ana Cristina Pinotti Pedro Ludovice, Bruno Pereira Valdigem, Nilton José Carneiro da Silva, Guilherme Fenelon

**Affiliations:** 1 Hospital Israelita Albert Einstein, São Paulo, SP, Brazil.; 2 Escola Paulista de Medicina, Universidade Federal de São Paulo, São Paulo, SP, Brazil.

**Keywords:** Monitoring, physiologic/instrumentation, Monitoring, physiologic/ methods, Arrhythmias, cardiac/diagnosis, Syncope

## Abstract

**Objective:**

To correlate arrhythmic symptoms with the presence of significant arrhythmias through the external event monitoring (web-loop).

**Methods:**

Between January and December 2011, the web-loop was connected to 112 patients (46% of them were women, mean age 52±21 years old). Specific arrhythmic symptoms were defined as palpitations, pre-syncope and syncope observed during the monitoring. Supraventricular tachycardia, atrial flutter or fibrillation, ventricular tachycardia, pauses greater than 2 seconds or advanced atrioventricular block were classified as significant arrhythmia. The association between symptoms and significant arrhythmias were analyzed.

**Results:**

The web-loop recorded arrhythmic symptoms in 74 (66%) patients. Of these, in only 14 (19%) patients the association between symptoms and significant cardiac arrhythmia was detected. Moreover, significant arrhythmia was found in 11 (9.8%) asymptomatic patients. There was no association between presence of major symptoms and significant cardiac arrhythmia (OR=0.57, CI95%: 0.21-1.57; p=0.23).

**Conclusion:**

We found no association between major symptoms and significant cardiac arrhythmia in patients submitted to event recorder monitoring. Event loop recorder was useful to elucidate cases of palpitations and syncope in symptomatic patients.

## INTRODUCTION

Palpitations, dizziness, pre-syncope and syncope are common complaints in physicians’ offices that might be related to cardiac arrhythmias. In some patients these symptoms can be triggered by stress and anxiety, but not associated with rhythmic disorders. The diagnosis and adequate treatment depend on electrocardiographic record during symptoms occurrence. However, this record is not always easy and in case of sporadic symptoms, the continuous electrocardiogram (ECG) record for 24 hours (Holter) might not be efficient.^(1)^

External event monitor is a device that records ECG intermittently when activated. It increases the accuracy of diagnosis when symptoms occur less frequently, *i.e., *weekly, monthly or yearly.^(2)^

The correlation of arrhythmic symptoms and electrocardiographic record of significant arrhythmias using external events monitoring, which is known as web-loop, is still not determined in our population.

## OBJECTIVE

To assess the association between specific symptoms (palpitations, pre-syncope and syncope) and identification of clinically significant arrhythmias through the web-loop, which is a specific type of external event monitor.

## METHODS

Between January and December 2011 we revised exams of 112 patients who received an external event monitor at an arrhythmia center in *Morumbi* and *Ibirapuera* unit of the *Hospital Israelita Albert Einstein*. This study was approved by the ethical and Research Committee (CAAE: 14097413.3.0000.0071) and the consent form was waived because it was a retrospective study.

External event monitor used was the Web Loop CW-10 (CardioWEB, São Paulo, Brazil). The device was connected to patient’s chest using two cables to collect the electrocardiographic sign. Patients were advised to keep the device as much as possible, removing it only to shower.

Web-loop system collected and transmitted automatically the electrocardiographic sign for 15 seconds every each 60 minutes. When the patient had symptoms such as palpitations, pre-syncope and syncope he/she would press the record button located at the bottom of the device. Another electrocardiographic record was also transmitted but lasting for 45 seconds, and 15 of them were recorded immediate before the system activation. Tracings were transmitted automatically using a GSM system (mobile signal) and a provider made available parts of the ECG collected through an internet homepage (https://looper.ecgweb.com.br).

Tracings transmitted were checked daily by the nursing team whose always called the patient when an event was recorded voluntary, and symptoms reported by the patient were documented. Medical team assessed tracings transmitted periodically and documented the electrocardiographic diagnosis. Standard duration of monitoring was 10 days, but when necessary it was stopped prematurely, and in case of significant arrhythmia record or if requested by the physician the monitoring was prolonged.

Specific symptoms were defined as palpitations, pre-syncope or syncope presented during monitoring. Significant arrhythmias were defined as paroxysmal supraventricular tachycardia, atrial flutter, atrial fibrillation, ventricular tachycardia, both supported (more than 30 seconds of length) and non-supported, besides pauses greater than 2 seconds or second and third degree atrioventricular block. Symptomatic arrhythmias were defined as any arrhythmia along with symptoms (significant, but also supraventricular and ventricular extrasystoles, isolated or matched).

Continuous variables were described in means ± standard deviations, and categorical variables were described in absolute and relative frequencies. The χ^2 ^test was used to assess association between presence of symptoms and significant arrhythmias. P<0.05 was considered as statistically significant.

## RESULTS

Of 112 patients who were assessed, 46% (n=51) were women. Patients’ mean age was 52±21 years with absolute values ranging between 13 days of life and 90 years (Table 1).

**Table 1:** Demographic characteristics of population monitored using the event monitor

Clinical characteristics	Results
Age, years	52±21
Women, n (%)	51 (46)
Systemic arterial hypertension, n (%)	33 (29.5)
*Diabetes mellitus, n (%) *	7 (6.2)
Previous myocardial infarction, n (%)	0
Previous stroke, n (%)	3 (2.7)

The event monitor was activated to record 118 events (each patient had more than one symptomatic event). In these 118 events, more frequent symptoms were palpitations (49.1%), followed by dizziness, pre-syncope and syncope (24.6%). Non-arrhythmic symptoms such as chest pain and dyspnea were seen in 26.3% of cases.

**Figure 1:**
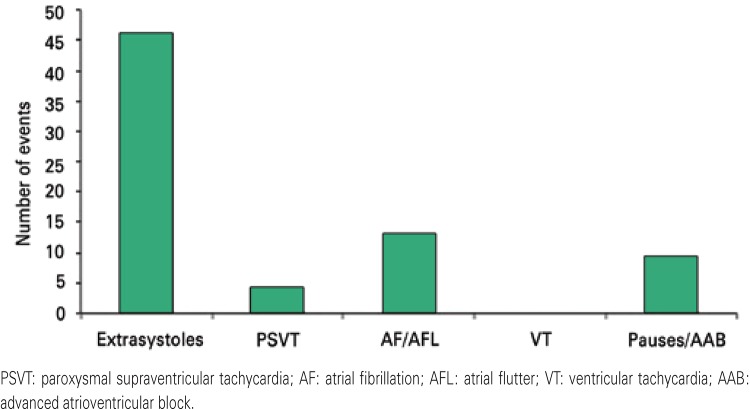
Types of arrhythmias detected through the event monitor

Among rhythm alterations detected, arrhythmias found were ventricular extrasystoles (n=46; 63.9%), atrial flutter and atrial fibrillation (n=13; 18.1%), pauses and second and third degree atrioventricular blocks (n=9; 12.5%) and supraventricular paroxysmal tachycardia (n=4; 5.5%), as shown in figure 1.

**Figure 2:**
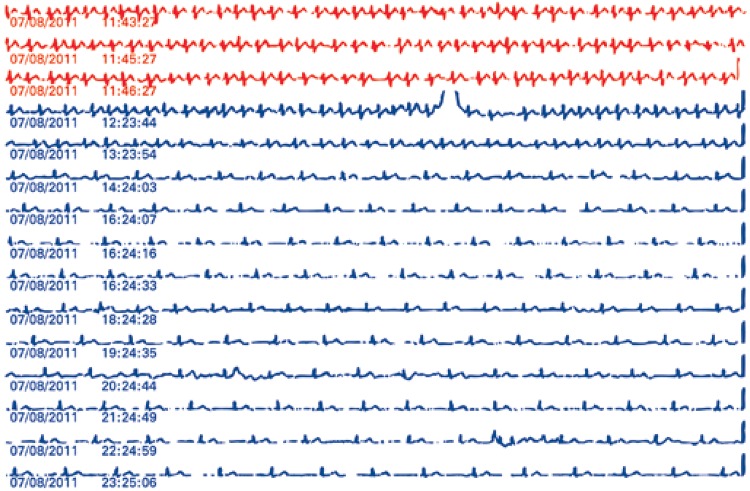
Tracing of web-loop during palpitation (diagnosis of atrial fibrillation) in a 57-year-old man. Red tracing represents symptoms record in electrocardiographic derivation at the moment of system activation (during 45 seconds). Blue tracing are automatic and periodic emissions of electrocardiogram in a derivation (lasting 15 seconds each)

**Figure 3:**
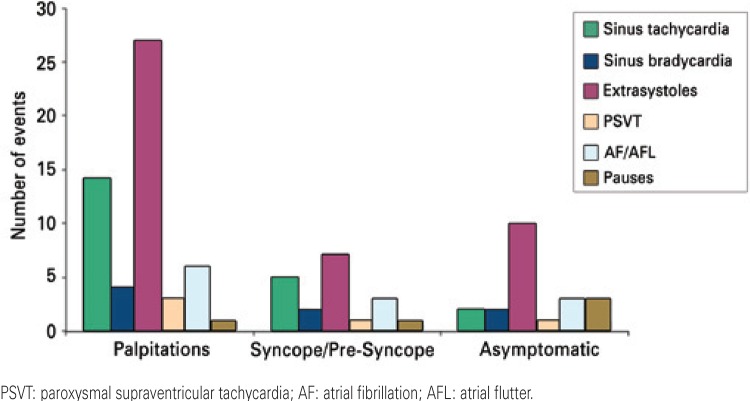
Types of rhythms and/or arrhythmias found according to each specific symptom reported

Palpitation was the most frequent symptom along with supraventricular and ventricular ectopics (n=27; 47%). The most detected significant arrhythmias during complaints of “palpitations” were atrial flutter and atrial fibrillation (n=6; 10.3%), as shown in figure 2. Symptoms as syncope, pre-syncope and dizziness occurred both during ventricular and supraventricular extrasystoles records (n=7; 24.1%), as well as in sinus tachycardia (n=5; 17.2%). Significant arrhythmias was most commonly detected in patients with symptoms of syncope, pre-syncope and dizziness as well as atrial flutter and atrial fibrillation (n=3; 10.3%), as shown in figure 3.

In 11 patients (9.8%) when there was no symptoms, the significant arrhythmia was detected – atrial flutter and atrial fibrillation (n=5), pauses (n=4) and supraventricular paroxysmal tachycardia (n=2).

Specific symptoms (palpitations, pre-syncope and syncope) were seen in 74 (66%) of monitored patients. However, the association between specific symptoms and significant arrhythmia was observed in only 14 patients (19%) (Table 2). There was no association between presence of symptoms and detection of significant arrhythmia (OR=0.57, confidence interval of 95% - CI 95%: 0.21-1, 57: p=0.23).

**Table 2:** Result of significant arrhythmia detection by events monitor

Specific symptoms	Significant arrhythmia n (%)	Significant without arrhythmia n (%)	Total n (%)
Presented symptoms	14 (12.5)	60 (53.5)	74 (66)
Absent symptoms	11 (9.8)	27 (24.2)	38 (34)

Total	25 (22.3)	87(77.7)	112 (100)

## DISCUSSION

Palpitations are responsible for 16% of complaints in physicians’ offices.^(3)^ The cardiac etiology is responsible for 43% of complaints, whereas psychiatric causes is responsible for 31% of cases.^(4)^ The correlation between symptom and tracing is essential to understand’ etiology of symptoms, so that long-term electrocardiographic monitoring appears as a diagnostic option.

Among clinical disease that lead patients to seek immediate health care, syncope is responsible to about 3% of admission at emergency service and about 1% of hospital admissions.^(5)^ Because mortality rate of syncope can reach up to 33% within one year in patients with structural heart disease,^(6)^ the adequate diagnostic clarification is fundamental and for this reason, exams such as ECG, echocardiography, effort test and 24-hour electrocardiography record (Holter) are recommended as the first steps.^(2)^

However, palpitations, syncope and pre-syncope of sporadic occurrence can be difficult to diagnose and also can lead to the need of using prolonged electrocardiography monitoring so that increasing the diagnosis sensibility and specificity. For this reason, loop memory internal and external event monitors (loop recorders) had gained relevance.^(7)^ Implantable monitors have a memory that can store electrocardiographic tracing of up to 40 minutes before activations and 2 minutes after it. These monitors can be easily implanted in the under patients’ skin and its battery last for 15 to 18 months.^(8)^ Therefore, implantable monitor is the most precisely way to investigate patients with infrequent symptoms (intervals between events greater than 1 month), and also because it presents more accuracy in the diagnosis for its monitoring for longer time periods. However, implantable devices have a higher cost and need a surgical procedure for implantation. In addition, post-operatory period can cause pain, risk of infection, and negative aesthetic issues in the implanted site.

External monitor, however, has disadvantages such as the need of electrodes attached to the skin that might cause irritation and need of discontinuity monitoring. Unfortunately, these monitors still not able to detect events automatically and they need to be activated by the patient or his/her caregiver when symptoms occur. This type of monitor constitutes a non-invasive method, less expensive and can be a cost-effective option mainly in those patients with low frequency of symptoms (weekly or monthly intervals between episodes).

In our study population we observed in 66% of patients with external event monitor, web-loop type monitor, the device was able to clarify arrhythmic symptoms, which is a result similar to data found in the literature using other types of event monitors.^(9)^ For palpitations the accuracy of diagnosis ranged between 66% and 75%, which is higher than the efficacy of the 24-hours Holter.^(10)^ And, to clarify symptoms of syncope and pre-syncope, the accuracy of external event monitor is low – about 25%. However, implantable event monitor is able to clarify between 35% and 88% of inexplicable syncope.^(11)^ Data found in our study reinforce that web-loop type monitor is useful to clarify symptoms of palpitations, but to clarify medical pictures of syncope, mainly those of sporadic occurrence, the implantable monitor seems to be more adequate.

Arrhythmias most often detected using the external event monitor were ventricular and supraventricular extrasystoles followed by supraventricular tachycardia. These findings agree with finding in studies with low-risk population.^(10)^ The fact that web-loop detected significant arrhythmias in patients who did not have arrhythmic symptoms raised our attention, and cases detected represented 9.8% of those investigated. Although web-loop was designed to detected symptomatic events, its transmission of random tracing automatically might be contributed for this finding. Such data suggest this method can have an important role in the diagnosis of asymptomatic arrhythmias, mainly in clinical pictures of paroxysmal atrial fibrillation - an arrhythmia that is associated with thromboembolic events.^(12)^ The improvement of web-loop by installation of mechanisms to detect automatically tachyarrhythmias would contribute for such indication.

In our study with patients submitted to heart rhythm monitoring by monitoring of events, symptoms of palpitations, pre-syncope or syncope were not associated to greater detection of significant arrhythmias. However, presence of symptoms in the absence of heart rhythm changes constituted useful clinical tool to exclude the possible cause of arrhythmia as an etiology of symptoms so that supporting the patient’s clinical management. External event monitor is useful to record ECG during arrhythmic symptoms and, although there is no high detection of atrial, ventricular tachyarrhythmias or atrioventricular blocks, the fact of recording the absence of arrhythmia is relevant and helps to guide the patient concerning the benign of his/her symptoms, and in this way to avoid the use of antiarrhythmic drugs or the need of invasive procedures to elucidate diagnostic symptoms.

This study is limited by the use of a retrospective designed based on collection of information which were recorded during exams (electrocardiographic tracing obtained and simplified questionnaires completed by patients just before monitoring begins). For this reason, relevant clinical information, *e.g.*, presence of structural hearth disease, use of antiarrhythmic drugs and history of psychiatric disease were not adequate documented. Therefore, to conduct a multivariate analysis adjusted to results was not possible. These information should be included in an future prospective study.

## CONCLUSION

The use of external event monitor, web-loop type monitor, did not show association between presence of specific symptoms and detection of significant arrhythmias in the studied population. This method of diagnosis seems to be importance to elucidate symptoms, especially, in case of no change in heart rhythm. External event monitoring constitutes useful clinical tool to exclude possible arrhythmia as etiology.
