# Supplementary material for: The use of external event monitoring (web-loop) in the elucidation of symptoms associated with arrhythmias in a general population
Source: Einstein (Sao Paulo). 2014 Jul-Sep;12(3):295–9. doi: 10.1590/S1679-45082014AO2939 (PMC4872938; doi:10.1590/S1679-45082014AO2939)
Supplement: Translation

# Translation: Utilização do monitor de eventos externo (*web-loop*) na identificação de sintomas associados a arritmias cardíacas em uma população geral

**Authors:** Hindalis Ballesteros Epifanio, Marcelo Katz, Melania Aparecida Borges, Alessandra da Graça Corrêa, Fátima Dumas Cintra, Rodrigo Leandro Grinberg, Ana Cristina Pinotti Pedro Ludovice, Bruno Pereira Valdigem, Nilton José Carneiro da Silva, Guilherme Fenelon

**Affiliations:** 1 Hospital Israelita Albert Einstein, São Paulo, SP, Brasil.; 2 Escola Paulista de Medicina, Universidade Federal de São Paulo, São Paulo, SP, Brasil.

**Keywords:** Monitorização fisiológica/instrumentação, Monitorização fisiológica/métodos, Arritmias cardíacas/diagnóstico, Síncope

## Abstract

**Objetivo:**

Correlacionar sintomas arrítmicos com a presença de arritmias significativas por meio do monitor de eventos externo (*web-loop*).

**Métodos:**

Entre janeiro e dezembro de 2011, o *web-loop* foi instalado em 112 pacientes (46% mulheres, 52±21 anos). Sintomas específicos foram definidos como palpitação, pré-síncope e síncope, presentes durante a monitorização. Arritmia significativa foi definida como taquicardia paroxística supraventricular, *flutter* e fibrilação atrial, taquicardia ventricular, pausas superiores a 2 segundos ou bloqueio atrioventricular avançado. A associação entre presença de sintomas e arritmias significativas foi avaliada.

**Resultados:**

O monitor de eventos registrou sintomas específicos em 74 (66%) pacientes, entretanto a associação entre sintomas específicos e arritmia significativa foi observada em apenas 14 (19%) deles. Em 11 pacientes (9,8%), foi detectada arritmia significativa na ausência de sintomas. Não houve associação entre a presença de sintomas e a detecção de arritmia significativa (OR=0,57, IC95%: 0,21-1,57; p=0,23).

**Conclusão:**

Em pacientes monitorizados pelo *web-loop*, não houve associação entre a presença de sintomas específicos e a detecção de arritmias significativas. O monitor de eventos pode ter importância na elucidação de sintomas de palpitações e síncope dos pacientes.

## INTRODUÇÃO

Sintomas de palpitação, tontura, pré-síncope e síncope são queixas comuns nos consultórios médicos e podem estar relacionados a arritmias cardíacas. Entretanto, alguns pacientes podem manifestar esses sintomas desencadeados por estresse e ansiedade, não associados com a presença de distúrbios do ritmo. O diagnóstico e o adequado tratamento dependem do registro eletrocardiográfico durante sua ocorrência. Contudo, esse registro nem sempre é fácil e, para sintomas de ocorrência esporádica, o registro contínuo do eletrocardiograma (ECG) por 24 horas (Holter) pode não ser eficaz.^(1)^

O monitor de eventos externo é um equipamento que realiza gravação intermitente do ECG quando acionado, aumentando a acurácia diagnóstica de sintomas cuja ocorrência é pouco frequente (semanal, mensal ou até anual).^(2)^

A correlação de sintomas arrítmicos com a documentação eletrocardiográfica de arritmias significativas, por meio do monitor de eventos externo, conhecido como *web-loop*, ainda não foi determinada em nossa população.

## OBJETIVO

Avaliar a associação entre sintomas específicos (palpitação, pré-síncope e síncope) e a detecção de arritmias clinicamente significativas utilizando o *web-loop*, um tipo específico de monitor de eventos externo*.*

## MÉTODOS

Foram revisados os exames de 112 pacientes consecutivos que instalaram o monitor de eventos externo no Centro de Arritmias do Hospital Israelita Albert Einstein, nas unidades Morumbi e Ibirapuera, entre janeiro e dezembro de 2011. Esse estudo foi aprovado pelo Comitê de Ética Institucional (CAAE: 14097413.3.0000.0071), sendo isento do Termo de Consentimento Livre e Esclarecido, por se tratar de estudo retrospectivo.

O monitor de eventos utilizado foi o *Web Loop* CW-10 (CardioWEB, São Paulo, Brasil). O gravador foi ligado ao tórax dos pacientes por meio de dois cabos para coletar o sinal eletrocardiográfico. Os pacientes foram orientados a permanecerem com o aparelho o maior tempo possível, retirando-o somente para o banho.

O sistema *web-loop* coletou e transmitiu automaticamente o sinal eletrocardiográfico, com duração de 15 segundos, a cada 60 minutos. Caso o paciente apresentasse sintomas como palpitação, pré-síncope ou síncope, ele devia acionar o botão de registro de eventos localizado na base do aparelho. Outro registro eletrocardiográfico foi, dessa forma, enviado, porém com 45 segundos de duração, 15 deles registrados imediatamente antes do acionamento do sistema. Os traçados foram transmitidos automaticamente, via sistema GSM (sinal dos celulares) a um provedor, que disponibilizou todos os trechos do ECG coletados em uma página da internet (https://looper.ecgweb.com.br).

Os traçados transmitidos foram diariamente checados pela equipe de enfermagem, que fez contato telefônico com o paciente sempre que houvesse o registro voluntário de eventos, sendo os sintomas referidos anotados. A equipe médica avaliou periodicamente os traçados transmitidos e registrou os diagnósticos eletrocardiográficos. A duração padrão de monitorização foi de 10 dias, podendo ser prematuramente interrompida, caso fosse registrado evento arrítmico significativo, ou prolongado, mediante solicitação médica.

Sintomas específicos foram definidos como palpitação, pré-síncope ou síncope presentes durante o período de monitorização. Arritmias significativas foram definidas como detecção de taquicardia paroxística supraventricular, *flutter* atrial, fibrilação atrial, taquicardia ventricular, tanto sustentados (mais de 30 segundos de duração) como não sustentados, além de pausas superiores a 2 segundos ou bloqueio atrioventricular de segundo e terceiro graus. Arritmias sintomáticas foram definidas como qualquer arritmia (significativas, mas também extrassístoles supraventriculares e ventriculares**,** isoladas ou pareadas) acompanhada por sintomas.

Variáveis contínuas foram descritas em média ± desvio padrão e variáveis categóricas na forma de frequências absolutas e relativas. O teste χ^2 ^foi utilizado para avaliar a associação entre presença de sintomas e arritmias significativas. O valor de p<0,05 foi considerado estatisticamente significativo.

## RESULTADOS

Dos 112 pacientes avaliados, 46% (n=51) eram do sexo feminino. A média da idade foi de 52±21 anos, com valores absolutos variando entre 13 dias de vida e 90 anos (Tabela 1).

**Tabela 1:** Características demográficas da população submetida à monitorização pelo monitor de eventos

Características clínicas	Resultados
Idade, anos	52±21
Sexo feminino, n (%)	51(46)
Hipertensão arterial sistêmica, n (%)	33 (29,5)
*Diabetes mellitus*, n (%)	7 (6,2)
Infarto do miocárdio prévio, n (%)	0
Acidente vascular cerebral prévio, n (%)	3 (2,7)

O monitor de eventos foi acionado para registrar 118 eventos (cada paciente pode ter tido mais de um evento sintomático). Nesses 118 eventos, os sintomas mais frequentes foram palpitações (49,1%), seguidas de tontura, pré-síncope e síncope (24,6%). Sintomas não arrítmicos, como dor torácica e dispneia, foram relatados em 26,3% dos casos.

Dentre as alterações do ritmo detectadas, as arritmias encontradas foram as extrassístoles ventriculares e supraventriculares (n=46; 63,9%), *flutter* e fibrilação atrial (n=13; 18,1%), pausas e bloqueios atrioventriculares de segundo e terceiro grau (n=9; 12,5%) e as taquicardias paroxísticas supraventriculares (n=4; 5,5%), conforme demonstrado figura 1.

**Figura 1:** Tipo de arritmias detectadas pelo monitor de eventos

O sintoma palpitação esteve relacionado mais frequentemente com a presença de ectopias ventriculares e supraventriculares (n=27; 47%). As arritmias significativas mais detectadas durante queixas de “palpitação” foram o *flutter* e a fibrilação atrial (n=6; 10,3%), conforme figura 2. Já os sintomas de síncope, pré-síncope e tontura ocorreram tanto durante o registro de extrassístoles ventriculares e supraventriculares (n=7; 24,1%), como de taquicardia sinusal (n=5; 17,2%). As arritmias significativas mais comumente detectadas nos pacientes com sintomas de síncope, pré-síncope e tontura também foram o *flutter* e a fibrilação atrial (n=3; 10,3%), como pode ser observado na figura 3.

**Figura 2:** Traçado do *web-loop* durante sintoma palpitação (diagnóstico de fibrilação atrial) em homem 57 anos. O traçado em vermelho representa registro do sintoma em uma derivação eletrocardiográfica no momento do acionamento do sistema (duração 45 segundos). O traçado em azul são as emissões automáticas e periódicas do eletrocardiograma em uma derivação (duração 15 segundos cada)

**Figura 3:** Tipos de ritmo e/ou arritmias encontrados conforme o sintoma específico referido

Em 11 pacientes (9,8%), foi detectada, na ausência de sintomas, arritmia significativa − fibrilação atrial e *flutter* atrial (n=5), pausas (n=4) e taquicardia paroxística supraventricular (n=2).

Os sintomas específicos (palpitações, pré-síncope e síncope) estiveram presentes em 74 (66%) pacientes monitorizados. No entanto, a associação entre sintomas específicos e arritmia significativa foi observada em apenas 14 (19%) pacientes (Tabela 2). Não houve associação entre a presença de sintomas e a detecção de arritmia significativa (OR=0,57, intervalo de confiança de 95% − IC95%: 0,21-1,57; p=0,23).

**Tabela 2:** Resultado da detecção de arritmias significativas pelo monitor de eventos

Sintomas específicos	Arritmia significativa n (%)	Sem arritmia significativa n (%)	Total n (%)
Presentes	14 (12,5)	60 (53,5)	74 (66)
Ausentes	11 (9,8)	27 (24,2)	38 (34)

Total	25 (22,3)	87(77,7)	112 (100)

## DISCUSSÃO

Palpitações são responsáveis por 16% das queixas em um consultório de clínica médica.^(3)^ A etiologia cardíaca é responsável por 43% das queixas, enquanto causas psiquiátricas chegam a 31% dos casos.^(4)^ A correlação do sintoma ao traçado é fundamental para o entendimento de sua etiologia e a monitorização eletrocardiográfica de longa duração pode ser uma opção diagnóstica.

Das doenças clínicas que precipitam a busca imediata por assistência médico-hospitalar, a síncope é responsável por cerca de 3% das consultas a pronto-socorros e por cerca de 1% das admissões hospitalares.^(5)^ Uma vez que sua taxa de mortalidade pode chegar até a 33% em 1 ano em pacientes com cardiopatia estrutural,^(6)^ adequado esclarecimento diagnostico é imprescindível, e, para isso, exames como ECG, ecocardiografia, teste de esforço e registro eletrocardiográfico de 24h (Holter) são os primeiros a serem realizados.^(2)^

Entretanto, palpitações, síncopes e pré-síncopes de ocorrência esporádica podem ter seu diagnóstico dificultado, o que leva à necessidade de utilização de métodos de monitorização eletrocardiográfica prolongada, aumentando a sensibilidade e a especificidade do diagnóstico. Foi então que os monitores de eventos com memória circular (*loop recorders*) internos e externos ganharam relevância.^(7)^ Os monitores implantáveis têm memória capaz de armazenar traçados eletrocardiográficos de até 40 minutos antes e 2 minutos após sua ativação. Podem ser facilmente implantados no subcutâneo e sua bateria tem duração entre 15 e 18 meses.^(8)^ Correspondem, portanto, à forma mais precisa de investigação para pacientes com sintomas infrequentes (intervalo entre eventos superiores a 1 mês), quando geram maior acurácia diagnóstica devido ao tempo estendido de monitorização. Entretanto, além do custo elevado do dispositivo, há necessidade de procedimento cirúrgico para implantação, cujo pós-operatório pode envolver dor, riscos de infecção, além de questões estéticas em relação ao sítio de implante.

O monitor externo, por sua vez, tem a desvantagem de necessitar de eletrodos colados à pele, podendo gerar intolerância e necessidade de descontinuação da monitorização. Não são, ainda, providos da função de detecção automática de eventos, necessitando do acionamento pelo paciente ou acompanhante na vigência de sintomas. Entretanto, é um método não invasivo e menos oneroso, podendo ser mais custo efetivo, principalmente naqueles pacientes com sintomas pouco frequentes (intervalo entre crises semanais ou mensais).

Na população estudada, observamos que, em 66% dos pacientes, foi possível esclarecer os sintomas arrítmicos pelo monitor de eventos externo do tipo *web-loop*, semelhante aos dados encontrados na literatura com outros tipos de monitores de eventos.^(9)^ Para sintomas de palpitações, a acurácia diagnóstica varia entre 66 a 75%, sendo superior a eficácia do Holter de 24 horas.^(10)^ Já para o esclarecimento de sintomas de síncope e pré-síncope, a acurácia do monitor de eventos externo é menor − em torno de 25%. Já o monitor de eventos implantável é capaz de esclarecer entre 35 a 88% dos casos de síncope inexplicada.^(11)^ Os dados encontrados no presente estudo reforçam que o monitor de eventos externo do tipo *web-loop* é útil para esclarecer sintomas de palpitações, mas, para esclarecimento de quadros de síncope, principalmente as de ocorrência esporádica, o monitor implantável parece mais apropriado.

As arritmias mais comuns detectadas por meio do monitor de eventos externo foram as extrassístoles ventriculares e supraventriculares, seguidas das taquiarritmias supraventriculares. Esses dados são compatíveis com o encontrado em estudos que envolvem populações de baixo risco.^(10)^ Chama a atenção o *web-loop* ter detectado arritmias significativas em pacientes que não tiveram sintomas arrítmicos, representando 9,8% dos casos estudados. Apesar de o *web-loop *ter sido concebido para detectar eventos sintomáticos, o fato de o mesmo enviar traçados aleatórios automáticos pode ter contribuído para tal achado. Esse dado sugere que esse método pode ter um papel no diagnóstico de arritmias assintomáticas, principalmente quadros de fibrilação atrial paroxística, arritmia que está associada a eventos tromboembólicos.^(12)^ O aprimoramento do *web-loop*, com instalação de mecanismos de detecção automática de taquiarritmias, poderá contribuir para essa indicação.

No presente estudo, nos pacientes submetidos à monitorização do ritmo cardíaco pelo monitor de eventos, os sintomas de palpitações, pré-síncope ou síncope não estiveram associados a uma maior detecção de arritmias significativas. Entretanto, a presença de sintomas na ausência de alteração do ritmo cardíaco constituiu-se em ferramenta clínica útil para excluir provável causa arrítmica como etiologia dos sintomas, auxiliando no manejo clínico do paciente. O monitor de eventos externo é útil para documentar o ECG durante sintomas arrítmicos e, ainda que não exista uma detecção elevada de taquiarritmias atriais, ventriculares ou bloqueios atrioventriculares, o fato de se documentar a ausência de arritmias é relevante e auxilia na orientação do paciente quanto à benignidade de seus sintomas, evitando o uso de drogas antiarrítmicas ou a realização de estudos invasivos para elucidação diagnóstica de sintomas.

Este estudo teve algumas limitações: trata-se de análise retrospectiva de dados com base na coleta de informações registradas durante o exame (traçados eletrocardiográficos obtidos e questionários simplificados preenchidos pelos pacientes imediatamente antes do início da monitorização). Dessa forma, informações clínicas relevantes, como, por exemplo: presença de cardiopatia estrutural, uso de medicações antiarrítmicas e história de doença psiquiátrica, não estavam devidamente documentadas. Por esse motivo, não foi possível realizar uma análise multivariada ajustada dos resultados. Num futuro estudo, prospectivo, essas informações devem ser contempladas.

## CONCLUSÃO

A utilização do monitor de eventos externo tipo *web-loop *demonstrou não haver associação entre a presença de sintomas específicos e a detecção de arritmias significativas na população avaliada. Esse método diagnóstico pode ter importância na elucidação dos sintomas, uma vez que estes, na ausência de alteração do ritmo cardíaco, constituem ferramenta clínica útil para excluir provável causa arrítmica como etiologia.
